# Parental Factors Influencing the Development of Early Childhood Caries in Developing Nations: A Systematic Review

**DOI:** 10.3389/fpubh.2018.00064

**Published:** 2018-03-16

**Authors:** Nayanjot Kaur Rai, Tamanna Tiwari

**Affiliations:** ^1^School of Dental Medicine, University of Colorado, Anschutz Medical Campus, Aurora, CO, United States

**Keywords:** dental caries, parents, child, risk factors, health knowledge attitudes practice, sociological factors

## Abstract

**Background:**

Early childhood caries (ECC) is one of the most prevalent and chronic conditions of childhood. Various factors including biological and dietary factors along with an overlay of parental social factors have been found to be associated with the progression of ECC. The objective of this systematic review is to synthesize available literature and to identify parent-level proximal and distal risk factors associated with the development of ECC in developing nations.

**Methods:**

Studies conducted in developing nations, published between 2005 and 2017 in English, that included children younger than 6 years and examined ECC were included. The outcome of interest were parental risk factors, which included parental knowledge, behavior, attitudes, sense of coherence (SOC), stress, socioeconomic status (SES), education, and breastfeeding duration. The studies were retrieved from MEDLINE, Ovid Medline, and PubMed.

**Results:**

The search yielded 325 studies, of which 18 were considered eligible for inclusion in this review. Ten studies found maternal education, and seven studies found parental education to be significantly associated with ECC. SES was significantly associated with ECC in 13 studies in the form of annual household income and occupation level. Four studies observed the significant association between oral health knowledge and attitudes with ECC, whereas only two studies found maternal attitude to be associated with ECC. Breastfeeding duration was a significant risk factor in four studies. One study each found significant associations of SOC, parental distress, and secondary smoke with ECC.

**Conclusion:**

To date, most of the researches done in developing countries have reported distal parental factors such as income and education being significant risk factors in caries development compared to proximal risk factors in low-income groups. Only a few studies analyzed the psychosocial and behavioral factors. Interventions could be designed to improve parental oral health knowledge and behaviors in these nations.

## Introduction

### Rationale

Early childhood caries (ECC) is considered as one of the most chronic and prevalent conditions of the childhood (1). It is a major public health problem that substantially impacts the life of individuals, families, and communities. It results in pain, debilitation in the oral and physical functionality of an individual, and harmful impact on the child’s growth rate and development, thereby decreasing the quality of life (2). Oral diseases pose large economic impact besides its personal and public implications, as they are the fourth most expensive to treat with an estimated public health expenditure of 5–10% (3–5). Developed countries have been studying the underlying causes and risk factors for ECC in various populations; however, similar literature is sparse for developing countries/nations, where the prevalence of ECC could be higher (6, 7). This report provides a review of recent work done in examining parental risk factors associated with ECC in developing nations.

The prevalence of ECC varies worldwide. Literature reports that the prevalence of ECC is between 1 and 12% in developed nations (8) compared to developing nations where the prevalence is as high as 70% (9). For example, some studies done in parts of India report the overall prevalence of ECC to be ranging between 27 and 42% (10–12). Similarly, various studies done in Brazil and China report the prevalence of ECC between 41 and 54% (13–15) and 40 and 78% (16–18) respectively.

Early childhood caries is a result of a multifaceted interaction of biological, genetic, and biochemical factors with an overlaying complexity of the social determinants of oral health (19). Although biological factors have been researched extensively, recent efforts have been done to evaluate social determinants of oral health and their influence on the development and progression of ECC (20). Such research has highlighted the distal, intermediate, and proximal level factors associated with ECC (21). Distal factors such as occupation, income, education, social class, and access to care are highly associated with ECC and maybe even termed as the root cause for disparities in ECC; however, most preventive intervention are targeted toward proximal factors, such as parent and child risk factors, because they are relatively easy to modify compared to distal determinants (21, 22). Proximal factors that mainly include oral health knowledge, behaviors, parental attitudes, and certain psychosocial factors have been reported to be some of the risk factors associated with ECC. There are several frameworks that discuss the interactions of distal, intermediate, and proximal factors related to ECC. One of the frameworks described by Patrick et al. (21) discussed this interaction and outlines the pathways through which political, social, cultural, and economic factors influence ECC (21). Another framework that focuses on proximal factors discusses how maternal education level, maternal beliefs, maternal locus of control (LOC), and family stress contribute to maternal psychological distress, which contribute to poor maternal oral health behaviors ultimately posing their children at higher risk for ECC (23). In addition, literature provides evidence of mother’s dietary preferences in playing a role in modeling her child’s dietary preferences, which could be either a protective or a risk factor for the child in developing ECC (24). Although there are fewer studies that have described the proximal parental risk factors associated with ECC, there is knowledge gap that exists about the impact of parental risk factors on the development of ECC.

### Objective

This current review aims to evaluate parental risk factors associated with the development of ECC studied in developing nations.

## Methods

### Study Design

In this review, we examined studies conducted in developing nations, published between 2005 and 2017. Studies published in English with full text available were included in this review. Developed countries have been studying the underlying root causes of ECC; however, the amount of literature studying the association between parental risk factors and ECC is sparse in developing nations. We have used the definition of developing countries/nations as described by the World Economic Situation and Prospects, United Nations (25).

### Data Sources and Data Extraction

Studies reporting parental risk factors in ECC were included as available in electronic databases, including MEDLINE *via* PubMed and Ovid Med and Web of Science.

### Search Strategy

Inclusion criteria for the search were as follows:

(1) Studies that used dental caries as an outcome measure. Most studies assessed dental caries by measuring decayed (d), missing (m), filled (f), surfaces (s), or teeth (t) rates; decayed (d), extracted (e), filled (f), or teeth (t) index; caries extending enamel (d_1_–d_2_) and dentin (d_3_) surfaces; decayed (d), filled (f), teeth (t) index, decayed teeth (dt), missing teeth (mt), filled teeth (ft) rates, or presence/absence of caries (i.e., dmft > 0). One study each measured dental caries as dft index, dt, mt, ft, and dmft indices.
(2) Studies that measured parental risk factors, including parental oral health knowledge, parental behavior, and attitudes; parental LOC, parental distress; socioeconomic status (SES) of the family in the form of annual household income and occupation level of both or either of the parents; education level of mother or both the parents; secondary smoking and breastfeeding duration.
(3) Studies that included children up to the age of 6 years.

The design of the studies to be included was not limited to any constraint, and both longitudinal and cross-sectional studies were taken into consideration. The qualitative research method was used to include studies in the review as no meta-analysis was conducted. The findings were synthesized using narrative synthesis.

**Figure 1:**
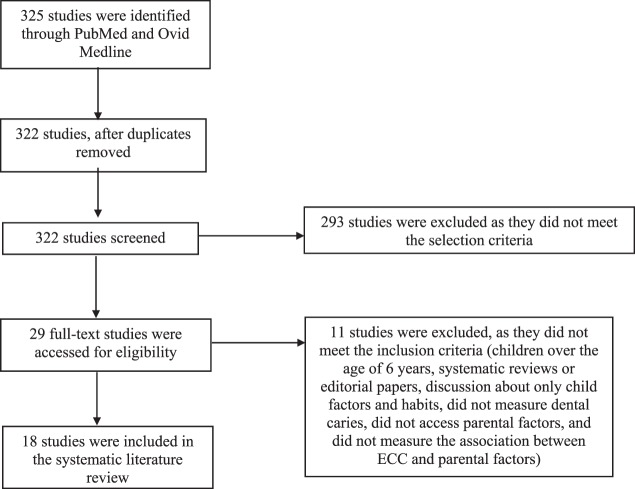
Flow diagram of the selection process for systematic review based on the PRISMA guidelines.

Initially, 325 studies were identified based on the selection criteria. The first selection process included a screening of the titles to remove any duplicates. As a result, three studies were removed. Second, abstracts were read for the 322 studies to access their suitability. The studies were excluded if they included children older than 6 years, were systematic reviews or editorials, involved discussion about only child factors and behaviors, did not measure dental caries, did not access parental risk factors (proximal and distal), and did not measure the association between ECC and parental risk factors. This resulted in the exclusion of 293 studies. Twenty-nine studies were then read in detail, of which 18 met the inclusion criteria and were included in the review and 11 were excluded. The flow diagram (Figure 1) explains the selection process for the studies included in the review. The studies to be included in the review were reviewed and finalized by both the authors and are presented in Table 1.

**Table 1:** Summary of studies included in review.

Study	Age; sample	Parental risk factors assessed	Caries index measured	Significant associations with ECC
**Asia**				

Sun et al.(16)	24–71 months; China	Supervised tooth brushing, parental education, oral health knowledge, attitudes, annual household income	dmfs	Supervised tooth brushing (*p* = 0.002), parental education (*p* = 0.033), oral health knowledge (*p* = 0.0001), attitudes (*p* = 0.0001), family’s income (*p* = 0.0001)

Jain et al. (26)	<71 months; India	Breastfeeding duration, mother’s education, both parent’s working	deft	Breastfed > 2 years (*p* < 0.05), mother’s education (*p* < 0.05), both parents working (*p* < 0.05)

Peltzer and Aroonsri (27)	<36 months; Thailand	Tobacco smoking by mother, parental education, household income	dmft and dmfs	Tobacco smoke (*p* ≤ 0.25), education (*p* < 0.001), household income (*p* < 0.05)

Sujlana and Pannu (28)	5 years; India	Parents’ education, SES, oral health knowledge, parental behavior, attitudes	dmft, dt, mt, and ft	Mothers’ education (*p* = 0.001), parents insisting to brush twice daily (*p* < 0.001)

Narang et al. (29)	3–6 years; India	SES, parental education	Diagnostic criteria according to Babeely K: 0 = NBS negative, 1 = mild, 2 = moderate, 3 = severe	Fathers lower education level (*p* < 0.001), mothers lower education level (*p* < 0.001), fathers lower occupation level (*p* < 0.001), mothers lower occupation level (*p* = 0.001), SES (*p* < 0.001)

Prakash et al. (30)	8–48 months; India	SES, mother’s education	deft	Low SES (*p* < 0.001), uneducated mothers (*p* < 0.05)

Menon et al. (31)	4–5 years; India	Parental distress, parental education, SES	dmft	Parenting distress (*p* = 0.05), SES (*p* = 0.02)

Wulaerhan et al. (17)	3–5 years; China	Oral health knowledge, parental education, SES	dmft	Parental education (*p* < 0.001), SES (*p* < 0.001)

Li et al. (18)	3–6 years; China	Education of parents, oral health knowledge, household income	dmfs and dmft	Parent education (*p* < 0.001), oral health knowledge (*p* < 0.001)

Zhou et al. (32)	2 years; China	Parent’s schooling, parent’s occupation, breastfeeding duration	dmft, VPI	Lower parent schooling (*p* = 0.003), occupation (*p* = 0.011), breastfeeding duration (*p* = 0.019)

**Africa**				

Folayan et al. (33)	6–71 months; Nigeria	Sociodemographic profile, oral health knowledge	dmft	Good oral health knowledge (*p* = 0.02)

Hamila (34)	1–3.5 years; Egypt	Maternal education, oral hygiene practices for mothers, employment status	dmft	Maternal education (*p* = 0.009), employment status (*p* ≤ 0.001), oral hygiene practice (*p* < 0.001)

Adeniyi et al. (35)	18–60 months; Nigeria	Oral health knowledge, Maternal attitude	dft index and OHI	Maternal attitude and OHI (*p* = 0.01), maternal attitude and dft (*p* = 0.001)

**Latin America**				

dos Santos Junior et al. (36)	3–4 years; Brazil	Family income	dmft	Family income (*p* = 0.009)

Borges et al. (15)	4–6 years; Brazil	Parental SES, parental education level	dmft	Low education (OR = 1.36), low family income (OR = 1.36)

Feldens et al. (37)	4 years; Brazil	Mothers’ education, family income, occupation level, breastfeeding duration	dmfs	Mothers’ education ≤ 8 years (*p* = 0.034), breastfeeding ≥ 7 times daily (*p* < 0.001)

Correa-Faria et al. (14)	3–5 years; Brazil	Monthly household income, parent’s schooling, breastfeeding duration	dmft	Place of residence (*p* = 0.004), mother’s schooling (*p* = 0.001), father’s schooling (*p* = 0.001), household income (*p* < 0.001), breastfeeding duration (*p* = 0.014)

Bonanato et al. (38)	5 years; Brazil	Parental social class, maternal SOC	dmft	Lower social class (*p* < 0.001), low SOC (*p* = 0.012)

*deft, decayed (d), extracted (e), filled (f), teeth (t) index; dft, decayed (d), filled (f), teeth (t) index; dmfs, decayed (d), missing (m), filled (f), surfaces (s); dmft, decayed (d), missing (m), filled (f), teeth (t); dt, decayed teeth; ft, filled teeth; mt, missing teeth; OHI, oral hygiene index; SES, socioeconomic status; SOC, sense of coherence; VPI, visible plaque index*.

The search terms used in PubMed to determine pertinent papers for the review were: *Dental Caries OR Dental Caries OR Dental Decay OR Carious Dentin OR dental caries OR dental AND caries OR dental caries OR carious AND dentins OR White Spots OR White Spot AND Child OR Child, Preschool OR child OR children OR Preschool Child OR Preschool Children AND Parents OR parents OR Parent OR Parenthood Status OR Parental Age OR Parental Ages AND Risk Factors OR Risk Factors OR Risk Factor AND Sociological Factors OR Sociological Factors OR Sociological Factor OR Sociological Characteristics OR Sociological Characteristic OR Social Attributes OR Social Attribute AND Health Knowledge, Attitudes, Practice OR Health Knowledge, Attitudes, Practice OR knowledge OR attitude OR attitudes OR behavior OR behaviors*.

### Risk of Bias Assessment and Quality Appraisal

According to the PRISMA guidelines (39), the studies included in this review were subjected to critical evaluation. To assess the quality of studies included in the review, we followed the model proposed by Hooley et al. (40) and addressed two methodological attributes: statistical analysis and sample characteristics. Studies were not removed from the review due to bias from methodological deficiencies, and so to address bias across the two methodological attributes of the studies, a ranking system was used (Table 2). The ranking criteria are discussed below.

**Table 2:** Quality assessment of studies across sample characteristics and statistical analysis.

Study	Sample rank	Analysis rank	Total score	Final rank
**Asia**				
Sun et al. (16)	3	2	5	C
Jain et al. (26)	2	2	4	B
Peltzer and Aroonsri (27)	1	2	3	A
Sujlana and Pannu (28)	3	1	4	B
Narang et al. (29)	4	2	6	D
Prakash et al. (30)	2	2	4	B
Menon et al. (31)	2	2	4	B
Wulaerhan et al. (17)	1	2	3	A
Li et al. (18)	1	2	3	A
Zhou et al. (32)	1	2	3	A

**Africa**				
Folayan et al. (33)	1	2	3	A
Hamila (34)	4	2	6	D
Adeniyi et al. (35)	1	2	3	A

**Latin America**				
dos Santos Junior et al. (36)	4	2	6	D
Borges et al. (15)	2	1	3	A
Feldens et al. (37)	4	1	5	C
Correa-Faria et al. (14)	4	2	6	D
Bonanato et al. (38)	2	2	4	B

*Sample rank: rank 1, stratified or cluster sampling of the population; rank 2, samples representative of cities or towns; rank 3, convenient and randomly selected samples; rank 4, non-random convenient samples*.

*Analysis rank: rank 1, high significance; rank 2, low significance*.

*Final rank: A, highest possible rank; D, lowest possible rank*.

Statistical analysis and data handling were evaluated based on the methods used to analyze the association between parental factors and ECC. The significance of the associations between the variable and ECC reported in the review based on the bivariate analysis without controlling for the potential confounding variables were classified of having low significance for the purpose of the review and were given the rank 2. However, the significance of the associations between the variable and ECC reported in the review based on the multivariate analysis while controlling for the potential confounding variables was classified of having high significance for the purpose of the review and were given the rank 1. Representativeness of the sample was evaluated, and the samples were classified as follows: rank 1—if the studies involved samples that were selected based on the stratified or cluster sampling of the population; rank 2—if the samples represented cities or towns; rank 3—if the samples were randomly selected and were convenient to access; and rank 4—if the samples were non-random and convenient to access. Each study was then ranked based on the quality across the two attributes. The highest possible score was 3 and the lowest was 6. They were then converted into A (highest possible rank) to D (lowest possible rank).

## Results

### Study Selection and Characteristics

Of the 18 studies included in the review, 7 were ranked A, 5 were ranked B, 2 were ranked C, and 4 were ranked D. Information about the quality of individual studies is presented in Table 2. These 18 studies were spread across countries in Asia, Africa, and Latin America. Fifteen studies were cross-sectional in design, two were prospective cohort and one was a case–control study. The summary of parental risk factors studied in the review is presented in Table 1. The parental risk factors are divided into the following categories: (1) distal factors—parental education level, and SES and (2) proximal factors—oral health knowledge and behaviors, psychosocial factors, and environmental factors.

Dental caries was assessed using different measures. Table 1 presents the different measures of dental caries used in the studies included in the review. Nine studies measured dmft index, and two studies measured dmfs, both dmft and dmfs, and deft index. One study each measured dental caries as dft index, dt, mt, ft, and dmft indices.

### Synthesized Findings

#### Distal Factors

##### Parental Education Level

Maternal education level was found to be significantly associated with ECC in 10 studies. Various formats were followed to measure the level of education. One study assessed maternal education level as no schooling, schooling till primary level, and schooling till high school (27). Another study compared mother’s schooling years of <12 to >12 years at the birth of their child (32). Also, mother’s education of ≤8 years was compared to >8 years in one of the studies (37). Another study assessed father’s education level as father’s schooling <8 years and ≥8 years (14). Another study compared father’s education at the university level, high school/primary school, or being illiterate (29).

The odds of maternal education level at the primary level and high school level were 2.06 and 2.21 times higher, respectively, compared to no schooling of the mother (*p* < 0.01) (27). The odds of having ECC were found to be twice for children whose mother’s schooling at child’s birth was <12 years compared to >12 years (32). In addition, the relative risk of developing ECC was found to be 1.5 times higher in children with mother’s education ≤8 years compared to mother’s education >8 years (*p* = 0.03) (37). It was seen that 21% of children had a dmft score ≥1 whose fathers completed university-level education compared to 89% of the children who went to high school, 44% who went to primary school, and 75% who were illiterate.

##### Socioeconomic Status

In addition to parental education, studies used SES as a predictor of ECC. It was found that families living in lower socioeconomic conditions had a high level of ECC in their children. Various methods were used to measure SES. Seven studies measured SES as lower annual/monthly household income (14–17, 27, 32, 36). One of the studies measured the association of lower family income with the presence of caries, rampant caries, and caries including non-cavitated lesions (15). Employment status in the form of parent occupation level and both parents working was measured in four studies (26, 29, 32, 34). Another study measured SES using Social Vulnerability Index (38). The index measured populations’ access to basic social services, including housing, schooling, income, jobs, legal assistance, health, and nutrition. The score ranged from 0 to 1, where a score closer to 1 indicated living in lower socioeconomic conditions.

Children of lower family income families were 1.3 times more likely to experience any caries, 1.4 times more likely to have rampant caries, and 1.2 more likely to experience non-cavitated lesions (15). Another study found that children from lower social class were 1.7 times more likely to have dental caries (*p* = 0.003) and 3.2 times more likely to have caries with pulp involvement (*p* = 0.001) compared to children belonging to upper class (*p* < 0.001) (38). Also, the prevalence of ECC was found to be significantly higher among children from low-income families in one of the studies (OR = 2.85) (17).

#### Proximal Factors

##### Parental Oral Health Knowledge and Oral Health Behaviors

Oral health knowledge and attitudes were assessed using a questionnaire. The questionnaire reflected the children and parents’ oral hygiene behaviors and parents’ attention to oral health. It measured knowledge on the use of fluoridated water and toothpaste, having sugary foods and drinks, the effectiveness of sealants, the frequency of tooth brushing, types of toothbrush used, and regular dental visits. Abiola Adeniyi et al. (35) measured maternal attitude toward dental health and assessed it based on mother’s attitude toward her own dental needs, her child’s dental needs, and toward prevention of ECC (35). Also, parental behaviors toward their children’s oral health were measured by accessing parental supervision with tooth brushing (16) and parental insistence on brushing twice daily (28). Oral hygiene practices for mothers and their children were also measured by one of the studies (34). In addition, breastfeeding duration was measured using different formats. One study compared children who were breastfed for more than 2 years to children breastfed up to 1 year (26). Another study compared breastfeeding ≥7 years daily or three to six times a day to once or twice a day (37).

Maternal oral health care knowledge was found to be significantly related to ECC in one study, which reported that children whose mothers had good oral health knowledge were less likely to have dental caries than children with mothers who had poor oral health knowledge (PR = −0.06, *p* = 0.02) (33). Another study found higher odds for developing ECC in children with lower parental oral health knowledge and attitudes (OR = 9.59, *p* = 0.0001) (16). In addition, dmfs and dmft scores were found to be higher (24) in children whose parents lack oral health care knowledge compared to children with parents having comprehensive oral health care knowledge (dmfs: lack = 8.32 ± 9.69, comprehensive = 6.74 ± 9.37; dmft: lack = 5.49 ± 4.77, comprehensive = 4.32 ± 4.68). Also, the odds of having better oral hygiene status (OR = 0.85, *p* < 0.0001) and dental caries status (OR = 0.85, *p* = 0.004) were found to be higher in children whose mothers had a good attitude toward their oral health (35).

It was reported that no parental supervision with tooth brushing (*p* = 0.002) was significantly associated with the development of ECC in children (16). Another study reported that in parents who insisted on brushing twice daily, their children were 1.8 times less likely to have ECC compared to parents who did not insist on brushing twice daily (*p* < 0.001) (28). In addition, oral hygiene practices for both the mothers (*p* < 0.001) and their children (*p* < 0.001) were significantly associated with ECC in one of the studies (34).

The duration of breastfeeding was found to be a significantly associated with the development of ECC in four studies (14, 26, 32, 37). It was reported that the average deft was highest in children who were breastfed for more than 2 years (*p* < 0.05, deft = 5.2) compared to the children who were breastfed only up to 1 year of age (deft = 4.65) (26). Also, it was found that the relative risk of developing ECC was 1.97 times higher in children who were breastfed ≥7 times daily and 2.04 higher who were breastfed three to six times a day compared to those breastfed once or twice a day (*p* < 0.001) (37).

##### Parental Psychosocial Factors

Mother’s SOC was found to be associated with ECC in one study (38). It was found that mothers with a lower SOC were 1.59 times more likely to have children with dt, 1.99 times more likely to have children with dental pulp exposure, and 1.85 times more likely to have children with ft compared to mothers with higher SOC.

A case–control study conducted by Menon et al. (31) found significantly higher mean parental distress scores (cases = 38.7 ± 12.34, controls = 36.63 ± 12.37, *p* = 0.02) and total parenting stress index scores (cases = 199.75 ± 59.56, controls = 189.64 ± 59.39, *p* = 0.02) among cases compared to controls (31). Statistically significant correlations were found between parenting distress and ECC (*r* = 0.78, *p* = 0.03) and between total parenting stress index and ECC (*r* = 0.80, *p* = 0.05).

##### Environmental Factors

Use of cigarette smoking by mothers was assessed when the child was 1 year old. It was observed that mothers who smoked had children with higher prevalence of S-ECC (27) compared to non-smoking mothers (*p* ≤ 0.25, prevalence of S-ECC = 44.1%).

## Discussion

### Summary of Main Findings

The findings of this review demonstrated that SES, parental education, oral health knowledge, and attitudes were associated with ECC in children. This report included studies that reviewed SES and parental education, parental psychosocial and environmental factors, and their correlations with ECC.

The education level of mothers was found to be associated with ECC in most of the studies included in the review. A strong correlation between maternal knowledge level and education has been reported by several researchers, which can ultimately influence the child’s oral health (19, 41). Also, mother’s schooling has been found to be an important oral health determinant interfacing with behavioral and psychosocial factors (32). In addition to the maternal education, father’s education has been associated with ECC in the literature, and a few studies included in this review used a similar measure (18). In addition to education, SES is a widely documented risk factor for ECC development (17). Social class and status of the family could determine the health beliefs and the perceived need for dental health care utilization of the family, which could be a factor that affects the oral health of the children and may lead to increased susceptibility to caries (31). It has been reported that families with low SES have significantly higher caries prevalence in their children compared to families living in high socioeconomic conditions (16). Similar associations were found between SES and dental caries in children in 13 studies included in the review.

Parental oral health behavior plays an important role in determining the oral health of their children (42). In the early years, parental oral health behaviors and feeding practices have been reported as the key risk factors associated with ECC (43). Children often follow their parents’ oral health behavior, which plays an essential part in the prevention of deciduous caries (16). According to American Academy of pediatric dentistry, parents should aid in tooth brushing of their children as younger children lack motivation and dexterity needed to brush their teeth (44, 45). Parental behaviors toward their child’s oral health including assistance with tooth brushing and motivation on brushing twice daily were found to be protective against ECC in three studies included in the review.

Maternal oral health knowledge, attitudes, and ECC do not share a simple cause-and-effect relationship (35). It is believed that parents having a good oral health knowledge tend to have a good attitude that may lead to following recommended oral health behavior on behalf of their children (35). In this review, we saw that children of mothers with high oral health knowledge and positive oral health attitudes had lower caries prevalence compared to children of mothers with poor oral health knowledge and attitudes.

Breast milk is known to contain immunological and nutritional components, which are essential for a child’s healthy development (46). Higher risk of ECC has been reported in children who were never breastfed or those who were breastfed for more than 24 months (47). Four studies included in the review found higher levels of caries in children with longer breastfeeding duration. Three studies had children who were breastfed for more than 1 year and were found to have higher caries index scores. One study, however, measured the frequency of breastfeeding at 1 year of age, which reported that children breastfed for three to six times had more caries compared to those breastfed once a day. However, these studies failed to report if the mothers received any anticipatory guidance related to breastfeeding behaviors, including oral hygiene following breastfeeding and frequency. Thus, including such information for new mothers might help to develop healthier breastfeeding practices.

Parental psychosocial factors are considered important in maintaining good oral health of their children. Factors such as SOC, oral health LOC, and health behaviors have been extensively studied in Western countries (6, 7, 48), and tailored interventions have been designed to improve the oral health knowledge and behaviors of parents, which ultimately reduces ECC. These factors have shown associations with the parental income/education and the community-related factors in several studies conducted in Western countries.

In looking at the bias in studies included in the review, we saw that only seven studies were ranked A based on the quality assessment across the two attributes. These studies used appropriate sampling and data analysis techniques, thereby minimizing the risk of bias. However, the studies ranked B, C, and D could be at a higher risk of bias due to either choosing bivariate analysis over multivariate analysis or random/non-random sampling techniques over stratified/cluster sampling techniques. The reliability and the validity of the study could have been compromised as the studies included in the review reported on small samples and were conducted in small regions of the developing nations, for example, India, China, and Brazil. These are large countries with significant variations in culture and language and health attitudes. Thus, the external validity of these studies could be undermined and not generalized to the outside population.

There were some limitations of this review. First, studies published in the English were taken into consideration for inclusion in the review. Hence, there is a possibility that some of the important work published in other languages was oversighted. Second, only MEDLINE was included to search the studies published in developing countries relating the review. Some developing countries might not have familiarity with MEDLINE or might not use MEDLINE to publish their work. Third, only electronically available studies were included in the review, and print studies were not taken into consideration.

## Conclusion

This review highlights the work of researchers in developing countries in studying parental risk factors as they relate to ECC. A higher number of researchers in these countries are studying the association of ECC with distal factors such as parental income and education factors compared to the proximal factors. These studies have shown a consistent association between low parental income and education to worst oral health outcomes in children. Thus, interventions could be designed that target to improve oral health knowledge and behavior in parents of low-income groups in developing nations. However, more research is needed to examine the influence of parental psychosocial factors, oral health knowledge, and behaviors on ECC development in children and to further the knowledge in this area.

## Author Contributions

NR and TT contributed to the design, acquisition, analysis, and interpretation of the data for the systematic review; drafted the work, revised it critically for important intellectual content, and approved the version to be published; and agreed to be accountable for all aspects of the work ensuring that the questions related to the accuracy or integrity of any part of the work are appropriately investigated and resolved.

## Conflict of Interest Statement

The research was conducted in the absence of any commercial or financial relationships that could be construed as a potential conflict of interest.
